# The implementation of an image‐guided system at a proton therapy center facility

**DOI:** 10.1002/acm2.14181

**Published:** 2023-10-14

**Authors:** Bijan Arjomandy, Ana Isabel Bejarano Buele, Neal Clinthorne, Milos Vujasevic, Basit Athar, James Deemer, Ahmad Alkhatib, Abrar Hussain

**Affiliations:** ^1^ Karmanos Cancer Institute at McLaren‐Flint McLaren Proton Therapy Center Flint Michigan USA; ^2^ Erlanger Baroness Hospital Chattanooga Tennessee USA; ^3^ Ehmet Health Plymouth Michigan USA

**Keywords:** image‐guided, Proton therapy, quality assurance

## Abstract

**Purpose:**

Pencil Beam Scanning (PBS) proton therapy has similar requirements on patient alignment to within 1 mm and 1‐degree accuracy as photon radiosurgery. This study describes general workflow, acceptance, and commissioning test procedures and their respective results for an independent robotic arm used for Image Guided Radiotherapy (IGRT) for a Proton Therapy System.

**Methods:**

The system is equipped with kV‐imaging techniques capable of orthogonal and Cone‐Beam Computed Tomography (CBCT) imaging modalities mounted on an independent robotic arm gantry attached to the ceiling. The imaging system is capable of 360‐degree rotation around patients to produce CBCT and kilovoltage orthogonal images. The imaging hardware is controlled by Ehmet Health XIS software, and MIM Software handles the image fusion and registration to an acceptable accuracy of ≤1‐mm shifts for patients’ alignment. The system was tested according to the requirements outlined in the American Association of Physicists in Medicine (AAPM) Task Group (TG) 142 and TG 179. The system tests included (1) safety, functionality, and connectivity, (2) mechanical testing, (3) image quality, (4) image registration, and (5) imaging dose. Additional tests included imaging gantry isocentricity with a laser tracker and collision‐avoiding system checks.

**Results:**

The orthogonal and volumetric imaging are comparable in quality to other commercially available On‐Board Imagers (OBI) systems. The resulting spatial resolution values were 1.8‐, 0.8‐, and 0.5‐Line Pairs per Millimeter (lp/mm) for orthogonal, full‐fan CBCT, and half‐fan CBCT, respectively. The image registration is accurate to within 1 mm and 1 degree. The data shows consistent imaging‐guided system performance with standard deviations in x, y, and z of 0.7, 0.8, and 0.7 mm, respectively.

**Conclusions:**

The system provides excellent image quality and performance, which can be used for IGRT. The proven accuracy of the x‐ray imaging and positioning system at McLaren Proton Therapy Center (MPTC) is 1 mm, making it suitable for proton therapy.

## 1 INTRODUCTION

**FIGURE 1:**
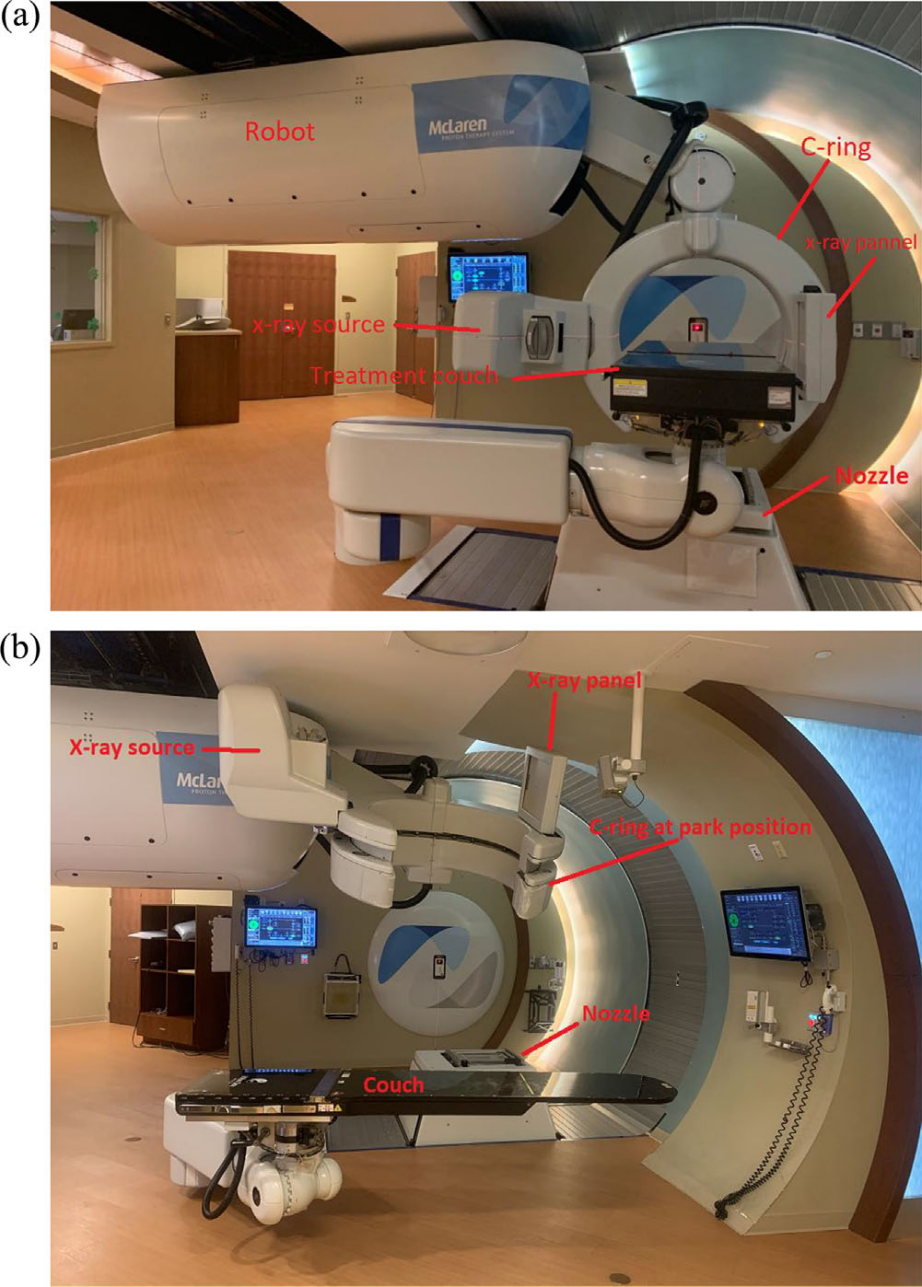
(a) MPTS treatment room with the imaging system in position for acquisition at couch 0 degrees. The c‐ring is positioned around the couch for image acquisition; the x‐ray panel and the source are on the left and right sides of the c‐ring, respectively . The nozzle is always positioned at the 180‐degree gantry angle during image acquisition. (b) The imaging system with the c‐ring in the park position and the couch in the load position. The x‐ray panel and source are on the left and right sides of the c‐ring, respectively. The nozzle is at 180 degrees. MPTS, McLaren Proton Therapy System.

Pencil Beam Scanning proton (PBS) therapy has similar requirements for precise image guidance for patient alignment as photon radiosurgery. The proton depth dose characteristics and range limitation of proton beams require that the patient be aligned to within 1 mm for optimal treatment.^1^, ^2^ The McLaren Proton Therapy System (MPTS) has an X‐ray Positioning System (XPS) with an x‐ray system capable of kilovoltage orthogonal and CBCT imaging modalities. The imaging hardware consists of a kV x‐ray generator source, tube assembly, and an amorphous silicon flat‐panel detector attached to a c‐ring gantry mounted on a robotic arm (Figure 1). The movements of the imaging arm and other parts (gantry and couch) in the treatment room are controlled and monitored by the Motion Control System (MCS) and a Collision Avoidance System (CAS) to prevent component collisions. The gantry must be at 180 degrees during the image acquisition. The clearance between the nozzle and the x‐ray panel is 4 cm at the gantry 180 degree. For full‐fan acquisition at the isocenter, the c‐ring only rotates 208 degree during CBCT acquisition to avoid x‐ray tube collision with the nozzle at 180 degree. Half‐fan acquisition, which requires 360‐degree rotation, is performed by shifting the patient position and the imager away from the nozzle by 20 cm while the gantry is set to 135‐degree to avoid a collision. The c‐ring only rotates 208 degrees during CBCT acquisition to avoid x‐ray tube collision with the nozzle. The MCS and CAS software were developed at MPTC.

The system acquires images under the control of the XIS software developed by Ehmet Health (Ehmet Health, Plymouth, Michigan, USA). The image fusion is handled by MIM software (MIM Software Inc., Beachwood, Ohio, USA), which accepts the acquired images and Digitally Reconstructed Radiographs (DRRs) from the planned field. The resulting couch correction coordinates are sent to MCS to be applied after imaging. Two IGRT modalities are currently supported: orthogonal imaging for orthogonal image pair and volumetric imaging for CBCT. The orthogonal images are used for craniospinal cases when multiple isocenters are used. All other patients’ alignments are performed using CBCT.

The MPTS is a unique proton delivery system that seamlessly integrates with Varian Aria OIS while adapting to different therapy workflow scenarios. The XIS has been designed and tested to provide a patient registration accuracy to within 1 mm at the room isocenter. The commissioning and Quality Assurance (QA) followed all aspects of kV and KV‐CBCT image quality and accuracy testing outlined in the Task Group (TG)‐142^3^ (tab. VI) and TG‐179^4^ (tab. II) recommendations. This study describes general workflow procedures, acceptance and commissioning test procedures, and their respective results.

## 2 MATERIALS AND METHODS

The imaging system hardware consists of an independent c‐ring gantry mounted on a robotic arm that holds a Varex RAD94 600 kHU x‐ray tube having 0.4‐ and 0.8‐mm focal spots capable of pulsed (CBCT) and continuous exposure (orthogonal radiographs) manufactured by North American Imaging (North American Imaging, Camarillo, California, USA) and a Varian 4030CB flat panel detector (Varian, Las Vegas, Nevada, USA). x‐Ray source and the detector mounted on the c‐ring arm with an open section of 68 cm, which rotates around the patient and acquires necessary projections. The SAD and SDD of the c‐ring are 100.3 and 148.9 cm, respectively. x‐Ray system positioning and gantry rotation around the patient are enabled by a series of electro‐mechanical controls that include Synqnet (RSI, Chicago, Illinois, USA) integration. The imaging gantry can travel through the room in a predefined path to position itself over the patient at supported couch angles (0, 180, and 270 degrees). In CBCT mode, the device is capable of full‐fan (195‐degree rotation) and half‐fan (360 degree rotation) image acquisition with the correct manual insertion of the appropriate collimator for Standard Field of View (SFOV) with 25 cm diameter transaxial x 17 cm axial and Large Field of View (LFOV) 46 cm diameter transaxial x 15 cm axial. The system incorporates bowtie and beam‐hardening filtration as well as a one‐dimensional antiscatter grid.

The XIS software orchestrates acquisition of orthogonal and CBCT images as well as interfaces with registration, MCS, treatment delivery, and OIS for offline review. Its user interface was designed with significant input from the therapy and physics teams at MPTS to make the complex task of patient alignment appear simple and to reduce potential setup errors (checks for DICOM plan file consistency, tight integration with MPTS workflow).

Registration can be performed with manual or automatic tools. The operator can choose among several preset imaging techniques and modify imaging parameters, such as kV and mAs, within a safe range if needed. Once acquired, the registration process follows after the user is satisfied with the image quality. MIM software performs the 2D‐2D orthogonal match by superimposing the acquired images on top of the planned DRRs in orthogonal mode or by superposing the CBCT on top of the planning CT. All the structure sets are visible on the planning CT for easy alignment. Once the rigid registration is done, the workflow in MIM ends and the calculated couch shifts are sent to the MCS for the operator to perform the couch correction for the correct patient setup.

**FIGURE 2:**
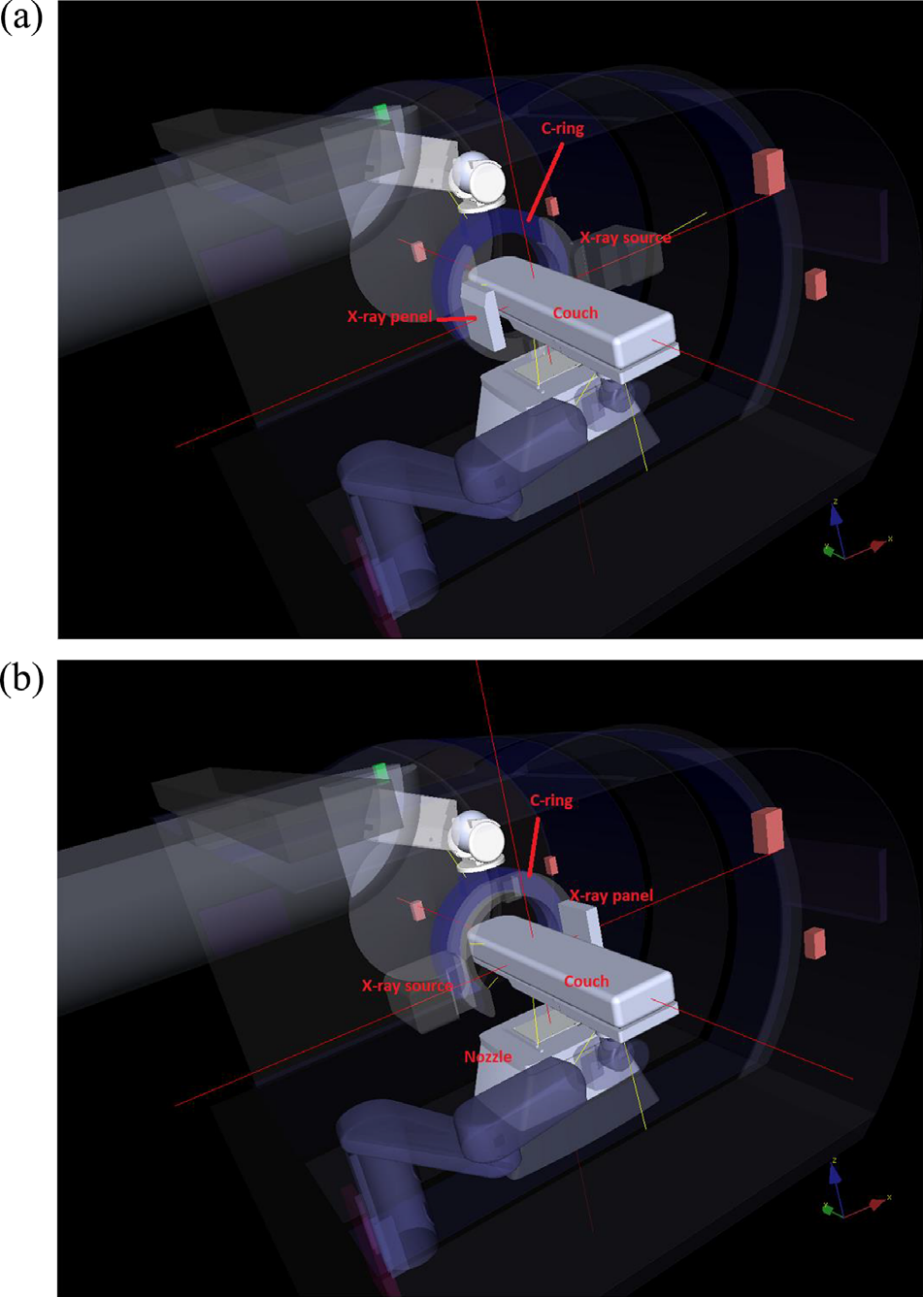
CAS real‐time monitoring model; (a) demonstrates the x‐ray panel on the right side for a 0‐degree couch while the source is on the left side (corresponding to the IEC 61217 fixed reference coordinate system (b) demonstrates the x‐ray on the left side of the 0‐degree couch while the source is on the right side. The CBCT ring is shown in blue. The patient collision envelope is shown atop the couch. The software detects the closest approach based on the modeled components slows down at a 25 cm distance, and stops the motions at 1 cm.

CAS monitors the imaging system, treatment couch, and gantry motions (Figure 2). The MCS and CAS increase workflow optimization by allowing the movement of several components simultaneously. It also halts the system when it approaches a state of possible collision with the patient or other system components. The system components and the patient as a long box template on top of the couch are incorporated into the software as a volumetric model. The CAS and MCS will not allow the user to acquire an image if the equipment does not meet the correct programmed configuration for the task.

### 2.1 Safety, functionality, and connectivity QA

**FIGURE 3:**
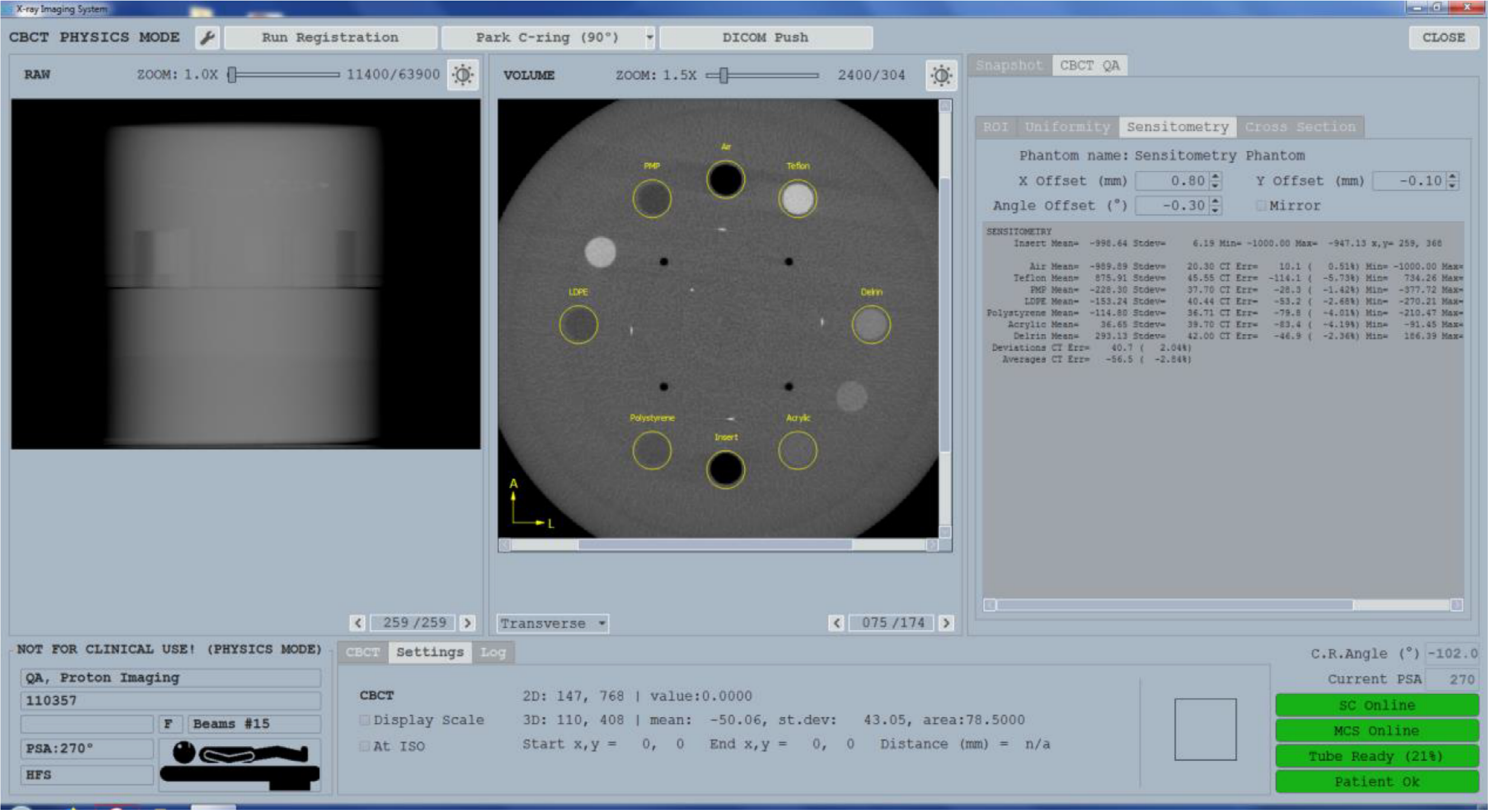
XIS CBCT physics QA mode for the sensitometry test. QA, quality assurance.

Due to the nature of the independent imaging gantry and connection to a third‐party machine and OIS, series of tests had to be conducted to assess the safety and connectivity of these systems. Redundant safety features allow the operation of the system to protect the patient and staff. These safety features are part of routine QA procedures, which include testing the collision pads on XPS and couch, door interlock, e‐stops, and x‐ray and beam on indicator. Functionality tests included the interconnectivity of motion enable buttons and software stop acquisition buttons. Connectivity consists of the interlocks between the CAS, MCS, X‐ray tube, treatment delivery console, and XIS's acquisition and reconstruction module. Software connectivity is checked easily by observing the indicators on the main XIS screen (Figure 3), which verifies the handshaking between the software.

### 2.2 Image registration and mechanical testing

Orthogonal pair (0 and 90 degs) and CBCT images are acquired for image registration and positioning purposes. The imaging device is routinely used to position the patients using online imaging with datasets from patient simulation. This requires the imaging and patient positioning systems to work together to achieve the correct shifts. The daily QA procedures verify the system's integrity by placing an Isocube phantom (Sun Nuclear, Nortfolk, Virginia, USA) on the couch top aligned with room lasers to its secondary crosshairs. The final registration shifts move the cube to align with the central crosshair with an accuracy of less than 1 mm. This is done for all supported couch positions.

### 2.3 Orthogonal image quality

For all orthogonal image quality tests, a TOR18FG phantom (Leeds Test Objectives, York, England) and an included 1‐mm copper plate were imaged at the isocenter. All default techniques were tested for spatial resolution, low contrast sensitivity, uniformity, and noise.

The spatial resolution test measures the ability of the imaging system to differentiate between two objects as they become smaller and adjacent to each other. The plate in the TOR18FG phantom contains 21 sets of line pairs with different spatial frequencies (0.5‐to‐5‐lp/mm). The resulting spatial resolution is given by identifying the sets of line pairs without artifacts or smearing. The ability of an x‐ray system to detect low‐contrast details depends on the noise and contrast loss levels due to scattering.^5^ The 18 discs in the TOR18FG phantom gradually decrease in contrast from a value of 0.167 to 0.009. The resulting low contrast sensitivity was given by the number of discs the user could see at the threshold of visibility. The resulting uniformity and noise were provided by the ratio and standard deviation of two regions of interest in a uniform area of the TOR18FG phantom.

### 2.4 CBCT image quality

A Catphan 604 phantom (The Phantom Laboratory, Salem, New York, USA) was imaged using all default CBCT protocols, including half and full‐fan techniques. All imaging processing was performed using the XIS software (Figure 3).

Geometric distortion was tested by measuring four markers spaced 50 mm apart on the module CTP604. Differences between nominal and measured values had a tolerance of <1 mm. The system's spatial resolution was verified using the Catphan phantom scan Section 1. Low contrast sensitivity was tested with the Catphan Phantom scan Section 3, which contains contrast targets (1.0%, 0.5%, and 0.3%) with varying diameters (2 to 15 mm). The resulting low contrast resolution is obtained by reconstructing the slice thickness to 5 mm and noting how many discs can be visualized. Scan slice thickness was measured according to the methods explained by the manufacturer of the Catphan phantom with the Geometry Module in the Catphan Phantom. Hounsfield Unit (HU) accuracy and linearity were tested by measuring the HU number of various inserts of different densities. The image uniformity test was done with the Catphan scan Section 4, designed to be within 2% (20 HU) of the density of water with standard scanning protocols. The original HU calibration was fit with a linear least‐squares fit.

### 2.5 Imaging dose

Measurements were performed with Computed Tomography Dose Index (CTDI) collimator masks that appropriately accounted for the detector size and the irradiated slice thickness (16.2 mm at the isocenter). The detector used for these measurements was a calibrated RadCal Accu‐Gold sensors and ion chamber (Radcal Corporation, Monrovia, California, USA). The Varian procedure described in “Dose in CT—On‐Board Imagers (OBI) Advanced Imagin”^6^ was used in calculating the CTDI dose.

## 3 RESULTS

### 3.1 Safety, functionality, and connectivity QA

The safety interlocks were tested during commissioning and as a part of daily and monthly checks with satisfactory results. The connectivity of the XIS with OIS and the proton delivery system has been verified daily with successful results. The efficacy of the CAS system was tested by intentionally bringing the system to a collision scenario, and the CAS interlock was successfully deployed the moment it detected collision.

### 3.2 Image registration and mechanical testing

**FIGURE 4:**
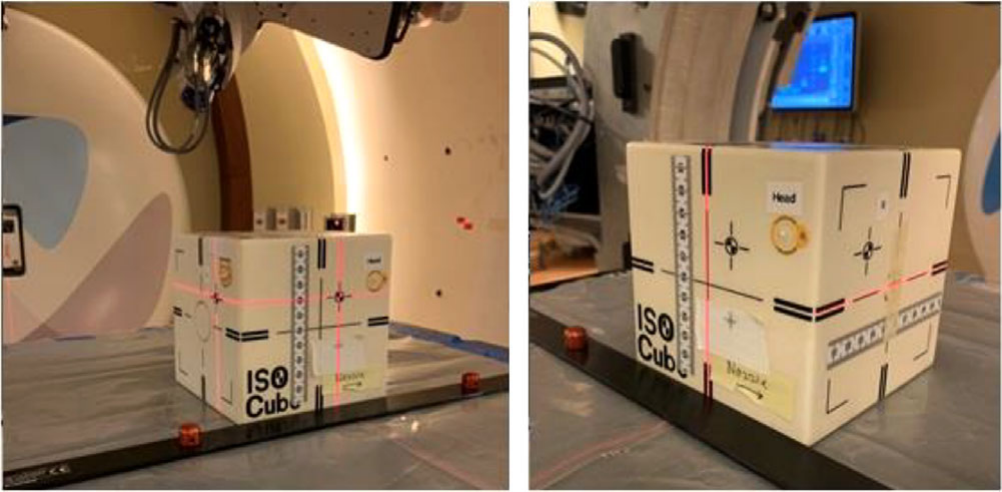
Starting position of the isocube on alternate crosshair relative to room isocenter (left). The final position of the cube relative to the room isocenter after applying couch shifts.

The off‐isocenter point with offsets 1.5, 2.5, and 2.0 cm in x, y, and z, respectively, on the cube was aligned with room lasers at the isocenter. After registration of images in either orthogonal or CBCT mode, the shifts were sent to MCS, which moved the couch and aligned the cube fixture at the isocenter (Figure 4). The cube position was verified by checking the central axis crosshairs with room lasers coincide. Manual and automatic tools were used to fuse and verify the images. The residual shifts are calculated based on the difference in the calculated shifts based on the image registration and the offset point. During the commissioning, a second set of images acquired to ensure proper alignment has been achieved after shifts were applied. The final alignment was also verified with room lasers as well. Sample results are summarized in Table 1. Deviations from expected values were below 1 mm and 1 degree. Differences in displacements in x‐y‐z were represented as a 3D displacement vector. The maximum deviation in the 3‐D displacement vector was 0.88 mm in orthogonal mode with the couch at 270 degrees. The minimum deviation in the 3‐D displacement vector was 0.34 mm in orthogonal mode with the couch at 180 degrees.

**TABLE 1:** Residual shifts of couch displacement for the isocube phantom.

	Displacement difference (mm/degrees)								
	Couch 0 degrees			Couch 180 degrees			Couch 270 degrees		
Modality	Ortho	CBCT	Difference (ortho‐CBCT)	Ortho	CBCT	Difference (ortho‐CBCT)	Ortho	CBCT	Difference (ortho‐CBCT)
X (mm)	−0.15	−0.09	0.06	0.28	−0.02	0.26	0.61	−0.04	0.57
Y (mm)	0.23	−0.51	−0.28	0.01	0.36	−0.35	0.63	−0.18	0.45
Z (mm)	0.29	0.17	0.12	0.2	0.5	−0.3	−0.02	−0.62	−0.6
Yaw (degree)	0	−0.163	−0.163	0	0	0	−0.2	−0.009	0.191
Pitch (degree)	0	−0.248	−0.248	0.6	0	0.6	0	−0.019	−0.019
Roll (degree)	0	−0.385	−0.385	0	0	0	0	−0.288	−0.288
3D displacement Vector XYZ (mm)	0.40	0.55		0.34	0.62		0.88	0.65	

For the object magnification factor test, the largest deviation on the measured points in the blade calibration fixture was −0.6 mm and −1.04 mm at 100 and 148.9 cm, respectively.

### 3.3 Orthogonal image quality

A summary of orthogonal image quality results is listed in Table 2. Various protocols were examined to test the system across different clinically used imaging parameters. The measurements showed the imaging system has a spatial resolution of 1.8 line pairs/mm across all protocols. The maximum number of visible discs was 17, with a contrast of 0.011, and the least value was 15 visible discs which corresponded to a contrast of 0.015. A score of 1 is given to a perfectly uniform image for image uniformity. The maximum and minimum uniformity score was found to be 1.064 and 1.005 corresponding to abdomen and thorax techniques, respectively.

**TABLE 2:** Summary of orthogonal image quality results.

Technique			Tests			
					Low contrast sensitivity	
Protocol	kVp	mAs	Spatial resolution (lp/mm)	Uniformity	Number of discs visible	Contrast of least visible disc
Abdomen	80	320	1.8	1.064	17	0.011
Pelvis‐Medium	75	100	1.8	1.004	16	0.013
Pelvis‐Large	75	160	1.8	1.009	16	0.013
Head	100	80	1.8	1.052	16	0.013
Thorax	75	100	1.8	1.005	15	0.015

### 3.4 CBCT image quality

A summary of CBCT image quality results for various techniques can be found in Table 3. The minimum deviation in the geometric distortion test was 0.01 mm, and the maximum was 0.82 mm. These deviations are within tolerance and can be the product of the reconstruction, image pixel density, and acquisition mode (half fan vs. full fan). As mentioned previously, the Catphan 604 protocols were used for half and full fan techniques. The low contrast resolution test was found to have positive results for the LFOV acquisition showing only the discs with 1% contrast. The reconstructed slice thickness agreed with the nominal thickness within 1 mm.

**TABLE 3:** Summary of CBCT image quality results.

Technique				Tests							
					Spatial resolution		Low contrast resolution				
Protocol	kVp	mA	ms	Geometric distortion Max Deviation (mm)	lp/cm	The gap size between lines (cm)	1%	0.50%	0.30%	Uniformity	Slice thickness (mm) (2.5 mm nominal)
Head‐Standard	100	25	32	0.82	8	0.053				1.59	
Head‐Low Dose	100	20	10	0.3	8	0.053				0.64	
Head‐High Quality	100	90	36	0.82	8	0.053				−0.30	
Pelvis	125	90	22	0.82	8	0.053				−0.16	
Pelvis (LFOV)	125	90	22	−0.01	5	0.088				−0.16	
Thorax	110	20	32	0.3	8	0.053				0.66	
Thorax (LFOV)	110	20	32	−0.01	5	0.088				0.32	
Catphan	100	40	25	0.82	8	0.053	0	0	0	0.73	2.5872
Catphan (LFOV)	100	40	25	−0.01	5	0.088	visible	0	0	0.21	2.5032

*NOTE*: The SFOV pixel size is 0.52 mm and LFOV pixel size is 0.93 mm with reconstructed image size of 512 × 512 voxels. The Catphan software was used for analysis of the parameters.

Abbreviations: LFOV, Large Field of View; SFOV, Standard Field of View.

### 3.5 Imaging dose

Table 4 reports the CTDI_w_ dose, a weighted average of individual edge measurements with the center. The highest CTDI_w_ dose was 20.2 mGy which belonged to the High‐quality head protocol (full‐fan mode), while the lowest dose was 1.4 mGy which belonged to the low‐dose head (full fan).

**TABLE 4:** Summary of CTDI_w_ for CBCT techniques.

Protocol	kV	mAs (total)	CTDI_w_ (mGy)	CTDI_w_/100mAs (mGy/100mAs)
High‐quality head	100	839	20.2	2.4
Standard head	100	202	5.1	2.4
Low dose head	100	52	1.4	2.8
Thorax (SFOV)	110	166	2.7	1.6
Thorax (LFOV)	110	307	3.3	1.1
Pelvis (SFOV)	125	513	11.7	2.3
Pelvis (LFOV)	125	948	14.3	1.5

Abbreviations: LFOV, Large Field of View; SFOV, Standard Field of View.

### 3.6 Observed clinical performance

**FIGURE 5:**
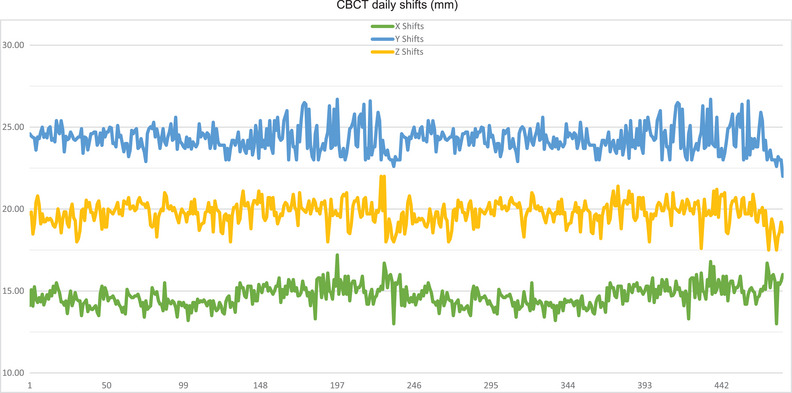
The variation in the robotic couch position was recorded for daily QA. The standard deviation in x, y, and z are 0.7, 0.8, and 0.7 mm, respectively. QA, quality assurance.

The imaging system has been tested daily and monthly since it was first used clinically in December 2018 to the present day. Daily and monthly tests and tolerances were set according to the baselines and recommendations outlined in TG‐142 with satisfactory results. Figure 5 shows sample data for daily QA reproducibility. The positions are the couch coordinates that are recorded daily after the shifts have been applied. The standard deviation in x, y, and z are 0.7, 0.8, and 0.7 mm, respectively. The imaging system has had an acceptable performance in clinical use.

## 4 DISCUSSION AND SUMMARY

The imaging quality is either the same or better than the existing imaging system used in other external beam and proton therapy centers.^7^, ^8^, ^9^ For example, Hua et al.^7^ examined a similar system to the MPTC system. He reported a residual translation shift of 0.02–0.76 mm compared to the MPTC system of 0.01–0.63 mm for three couch angles. Hua et al. have also reported CTDI in the range of 1.9–11.5 mGy for their system compared to the MPTC with CTDI of 1.4‐11.7 mGy for mAs less than 500. Chan et al.^8^ have reported the spatial resolutions for CBCT imaging systems ranging in 0.8–1.1, 0.8–1.0, and 0.4–0.5 lp/mm for Varian, Elekta, and Siemens, respectively, compared with MPTC range of 0.53–0.88 lp/mm for CBCT. Yoo et al.^9^ have reported spatial resolution for the OBI system of Varian 2100 EX to be 1.25–1.16 lp/mm.

The MPTC imaging system can perform CBCT and 2D radiographic imaging techniques. While the MPTC current calibration is done for only three couch angles, it has the potential to image the patients at any angle between 0 to 180 degrees. However, some existing imaging systems with fixed panel configurations attached to the gantry have limited capability. For example, the MPTC imaging system can image patients for vertex fields. However, there are some limitations when the arms on the wing board extend beyond the CAS model. Therapists routinely perform virtual sims on the first day to ensure the equipment clears the patients. Its isocenter is located in the middle of the couch, which is an issue for large patients and may have the possibility of collision with the patient. Since it is an independent system, it requires moving from the park to the image position, which increases the overall treatment time when left and right‐side treatments are needed, which is typically require an additional 10 min to treat two fields proton prostate case as compared to a conventional treatment.

Our quality assurance program follows guidelines from TG‐142 while accommodating additional testing requested by in‐house engineering. These tests were used to measure the imaging system's performance before and during clinical use. The frequency of these tests has allowed us to prove that the system is reliable and stable mechanically and in terms of image quality. We have found the system is suitable for the proton therapy environment. The proven accuracy of the imaging and positioning system at MPTC is within 1 mm, which is similar to those systems for SRS/SBRT systems.

## AUTHOR CONTRIBUTIONS

Bijan Arjomandy: Development of workflow, testing, verification, and manuscript author (physicist). Basit Athar: Testing and verification of the system and workflow (physicist). James Deemer: developer for MCS and hardware component of robotic arm for x‐ray and couch (Engineer). Ahmad Alkhatib: Data collection and analysis (physicist). Abrar Hussain: Testing and verification of imaging system (physicist). Ana Isabel Bejarano Buele: imaging protocol development, image quality testing, accuracy testing (physicist). Neal Clinthorne: XIS software developer and x‐ray hardware testing (Engineer). Milos Vujasevic : XiS software developer and x‐ray and workflow testing (engineer).

## CONFLICT OF INTEREST STATEMENT

The authors declare no conflicts of interest.
